# The Physique of Schoolboys

**Published:** 1898-11-26

**Authors:** 


### The Hospital

A Journal of

The Medical Sciences and Hospital Administration.

Vol. XXV.—No. 635.] Edited by Sir Henry Burdett, K.C.B., and Solomon C. Smith, M.D., M.R.C.P. [Nov. 26, 1898.

### The Physique of Schoolboys.

A good deal of public attention has been directed during the last few weeks to a question which has been quite correctly described as one of national importance, namely, the physical condition of boys among the middle and upper classes of society. The subject was brought under notice by a letter signed "M.D.," which appeared in the *Times* paper early in November, and which described the results of a careful examination of the health and development of a hundred boys, between the ages of thirteen and fifteen, taken without selection as they presented themselves on their first arrival as pupils at a great public school. The results were of a character to justify grave doubts with regard to the prevailing methods of feeding and of educating children, especially in their earlier years; for, in the cases in question, we are left wholly without guidance on the important question as to whether the defects discovered were due to mismanagement in the nursery, or to the conditions of life in preparatory schools. According to "M.D." the hundred boys were all assumed by their parents to be fair average specimens of their class. Omitting from consideration what he calls "minor imperfections," a phrase which includes a tendency to chilblains, defective teeth, and "many other failings," he found that thirty-nine boys were below the average in height, and fifty-three in weight. Sixty-eight were below the average in chest measurement, and sixty-three were the subjects of "deformities," such as lateral curvature of the spine, pigeon-breast, knock-knees, or flat-foot. Twenty had defective sight, nine had defective hearing, one an "abnormal growth" (its nature not mentioned), one was colour-blind, two had "heart disease," two were ruptured, fourteen had varicocele, and twenty-two albuminuria. It will be seen at once that, with regard to many of these conditions, the information given is not sufficient to afford material for any opinion as to their gravity, or as to the degree in which they were likely to constitute hindrances to the pursuit of various avocations in life; neither are we told in what proportion individual boys were the subjects of multiple defects. Notwithstanding these omissions, the catalogue fully justifies a somewhat anxious inquiry with regard to the degree in which the bodily wants of growing boys are habitually supplied by the methods of treatment which ordinarily prevail in families and in preparatory schools.

It must not be forgotten, in the first place, that parents, as a rule, are notoriously optimistic with reference to their offspring, whose qualities, both mental and physical, they are apt to appraise under the influence of feelings bearing some resemblance to conceit. The forgotten author of the fable about the owl and the eagle wrote from a profound knowledge of human nature. The old birds struck up a close friendship, and undertook to preserve and defend each other's young. "But I don't know your children by sight," observed the eagle. "You cannot mistake them," replied the owl, and proceeded to describe a group of nestlings among whom all sorts of beauties were combined in the most exquisite harmony. The friends separated, and, the next day, the eagle, finding three or four hideous little half-fledged scarecrows in a hole in a tree, ate them up, not only without ceremony, but without suspicion. Among parents of whom the owl may be taken as the type, there is either an unconsciousness of facts, or an unwillingness to look them in the face; and hence defects, which might to some extent be remedied during youth, are suffered to produce their consequences unchecked. An example of this is often furnished by the faulty positions of body which are constantly assumed under the influence of defective sight, and which may easily lead to spinal distortion. The defective sight could usually be improved by spectacles, and the faulty positions by care; but it frequently happens that both remain unnoticed, until permanent mischief has been produced, or until the children fall, for the first time, under the observation of some independent person. The family doctor could not be asked to fulfil a more important function than the systematic observation of nurseries with reference to the growth, general development, and physical condition of the inmates, with a view to the early correction either of bad habits or of the causes which produce them. Among other matters calling for such supervision, a prominent place should be given to food, which, in order to meet the special conditions of the growing period of life, should be much more carefully considered than as a rule it is. Parents can hardly be expected to understand the differences between the requirements of the child and those of the adult; a difference largely of proportion between the different elements of nutrition. Twenty-two per cent. of cases of albuminuria, in boys supposed to be healthy, can scarcely be due to anything else than an excessive ingestion of a kind of aliment which is not required for the support of the system, and is carried out as waste or redundant material by the kidneys. Less meat, and more oatmeal porridge and boiled milk, would probably prove an effective remedy.

